# Prime Editing: Mechanistic Insights and DNA Repair Modulation

**DOI:** 10.3390/cells14040277

**Published:** 2025-02-13

**Authors:** Astrid Mentani, Marcello Maresca, Anna Shiriaeva

**Affiliations:** Genome Engineering, Discovery Science, BioPharmaceuticals R&D, AstraZeneca, 43183 Mölndal, Sweden; marcello.maresca@astrazeneca.com

**Keywords:** Cas9, prime editing, DNA repair mechanisms, DNA repair modulation, homologous recombination, non-homologous end joining, microhomology-mediated end joining, single-strand annealing

## Abstract

Prime editing is a genome editing technique that allows precise modifications of cellular DNA without relying on donor DNA templates. Recently, several different prime editor proteins have been published in the literature, relying on single- or double-strand breaks. When prime editing occurs, the DNA undergoes one of several DNA repair pathways, and these processes can be modulated with the use of inhibitors. Firstly, this review provides an overview of several DNA repair mechanisms and their modulation by known inhibitors. In addition, we summarize different published prime editors and provide a comprehensive overview of associated DNA repair mechanisms. Finally, we discuss the delivery and safety aspects of prime editing.

## 1. Introduction

For many decades, scientists have aspired to introduce targeted, precise changes into the human genome. With the development of zinc finger nucleases (ZFNs) and transcription activator-like effector nucleases (TALENs), it became evident that this dream could become a reality [1,2]. However, the breakthrough occurred only after 2012, when the mechanism of CRISPR-Cas-mediated immunity was discovered in bacteria *Streptococcus pyogenes* and *Streptococcus thermophilus* [3,4]. The CRISPR-Cas system in these bacteria (later classified as type II-A) is an example of the diverse prokaryotic CRISPR-Cas systems, which include at least six types and thirty-three subtypes [5]. Cas9 is central to the mechanism of the type II CRISPR-Cas immunity. This protein binds to a short CRISPR RNA (crRNA) molecule, which contains a 20 nt sequence originating from a mobile genetic element. Guided by this sequence, Cas9 finds a complementary target in the DNA of an invading bacteriophage or plasmid and introduces a double-strand break (DSB) [3,4]. In bacteria, crRNA functions in tandem with another RNA molecule called tracrRNA. Jinek et al. demonstrated that crRNA and tracrRNA can be fused into a single guide RNA (sgRNA), providing researchers with a simple, programmable, two-component Cas9-sgRNA tool for the targeted introduction of DSBs into a genome of virtually any organism [3]. 

Knocking out genes using Cas9-sgRNA has already proven to be an efficient therapeutic strategy. Following the successful results of ongoing clinical trials [6,7,8], the first CRISPR-Cas9 knockout-based therapy, Casgevy (exagamglogene autotemcel), received regulatory approval for the treatment of transfusion-dependent β-thalassemia and sickle cell disease in 2023 [9]. Although Cas9-mediated knockouts can be relatively easily established, reverting pathogenic variants to a wild-type sequence is much more challenging due to several competing double-strand break repair (DSBR) pathways present in human cells. Numerous studies have identified key proteins involved in DNA repair pathways. Using this knowledge, it is possible to modulate the cellular DNA damage response in favor of a desired editing outcome. In the first part of this review, we provide an overview of DSBR in human cells and possible ways to promote the desired editing outcome. In the second part, we shift our focus to prime editing (PE)—an alternative gene editing strategy relying on a Cas9 nickase instead of a nuclease. We then discuss how the method has evolved in recent years and review the DNA repair factors involved with a focus on mismatch repair proteins.

Finally, we discuss the use of Cas9 nuclease in PE, the manipulation of DSBR to promote precise Cas9 nuclease-mediated PE, various delivery methods and the safety of this approach.

## 2. DSBR Pathways and Genome Editing Mediated by Homology-Directed Repair

### 2.1. DSBR in Human Cells

**Figure 1:**
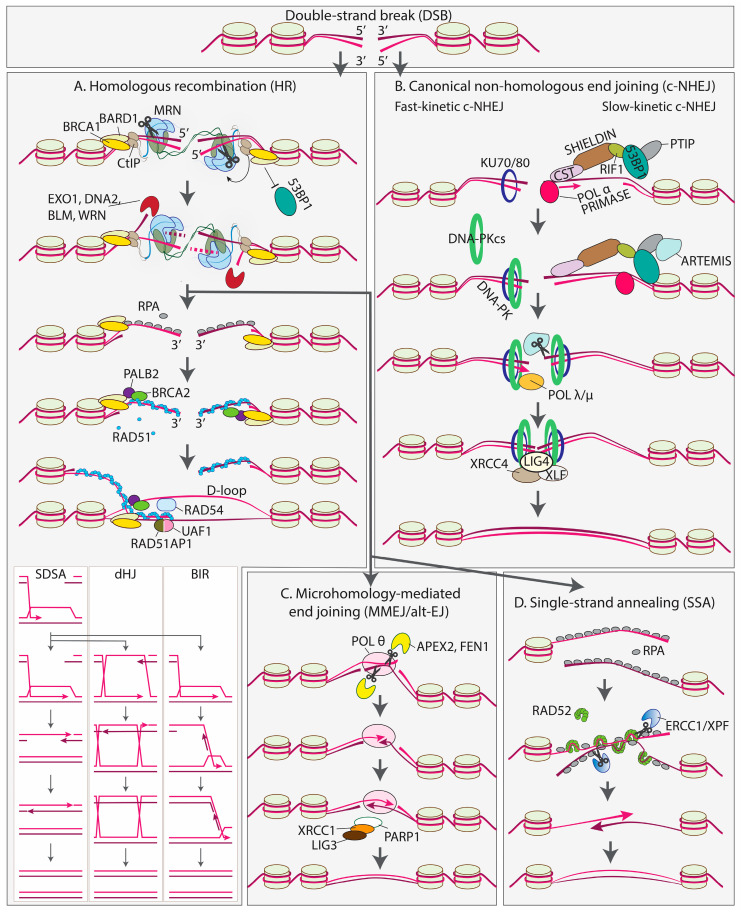
Double-strand break (DSB) repair pathways in human cells. (**A**) Homologous recombination (HR). A DSB is recognized by the MRN complex. Activated by CtIP, the MRN complex incises the 5′-terminated strand at some distance from the DSB and degrades the 5′-terminal part due to MRE11 3′-5′ exonuclease activity. Such ‘short-range resection’ is followed by ‘long-range resection’ performed by EXO1 and DNA2 exonucleases and BLM and WRN helicases. The BRCA1-BARD1 complex recognizes histone modifications specific to DSBs, stimulates resection, prevents classical non-homologus end joining (c-NHEJ) factor 53BP1 from loading onto the chromatin and recruits PALB2-BRCA2. RPA binds to the generated 3′ overhang. PALB2-BRCA2 stimulates the replacement of RPA with RAD51 recombinase. The RAD51 filament invades a homologous donor sequence, resulting in the formation of a displacement loop (D-loop) with the assistance of RAD54, PALB2-BRCA2, BRCA1-BARD1 and RAD51AP1-UAF1. The 3′ end is then extended by a DNA polymerase. The subsequent process is subdivided into synthesis-dependent strand annealing (SDSA), a double Holliday junction pathway (dHJ) and break-induced replication (BIR) depending on the presence of one or two ends and the interaction between the ends and the donor. (**B**) c-NHEJ. Depending on the complexity of the ends, c-NHEJ proceeds with fast or slow kinetics. Fast-kinetic c-NHEJ starts with the binding of the KU70/80 heterodimer to a blunt end or an end with a relatively short overhang. KU70/80 activates the DNA-dependent protein kinase catalytic subunit (DNA-PKcs), and, together, they form DNA-PK. In the case of slow-kinetic c-NHEJ, additional factors are necessary to counteract end resection and prepare the end for ligation. 53BP1 recognizes histone modifications specific to DSBs. This leads to the recruitment of additional factors: RIF1, SHIELDIN, CST, POL α/primase, PTIP and ARTEMIS. POL α/primase fills the 3′ overhangs. ARTEMIS nuclease removes the 3′ overhangs (or other overhangs if present). DNA POL λ or μ also contributes to the generation of compatible ends. The subsequent ligation is performed by the XRCC4-LIG4/XLF complex. (**C**) Following end resection, a DSB may be repaired via microhomology-mediated end joining (MMEJ), also called alternative end joining (alt-EJ). DNA POL θ promotes the annealing of the two 3′ ends due to microhomologies which are several nucleotides long. APEX2 and FEN1 remove 3′ flaps, preparing 3′ ends to be extended by POL θ. Single-strand breaks are ligated by LIG3/XRCC1/PARP1. (**D**) Single-strand annealing (SSA) is associated with hyper-resection of 5′ ends. The two 3′ ends are annealed through an extensive homology region mediated by RAD52 and RPA. 3′ flaps are removed by the ERCC1/XPF endonuclease. The process is finalized by gap filling and ligation, although it is not clear which DNA polymerase and DNA ligase are involved.

A DSB in human cells is repaired via one of four DSBR pathways, called homologous recombination (HR), canonical or classical non-homologous end joining (c-NHEJ), microhomology-mediated end joining (MMEJ, also called alt-EJ) and single-strand annealing (SSA) (Figure 1) [10,11,12,13,14]. HR can be further divided into three subpathways: the double Holliday junction pathway (dHJ), break-induced replication (BIR) and synthesis-dependent strand annealing (SDSA). In addition to HR, a related term, homology-directed repair (HDR), is often used in the literature. While some authors use this term interchangeably with HR, others define HR as a broader term relative to HDR or vice versa. Throughout this review, we will use ‘HR’ as a term referring to the mechanisms of DSBR and ‘HDR’ as a term describing any known or unknown homology-directed pathway for gene editing with exogeneous templates.

### 2.2. Homologous Recombination (HR)

HR is a DSBR pathway in which a homologous sequence is used as a template to extend one or both 3′ ends of a broken chromosome such that they gain sufficient homology between the two ends (Figure 1A). This allows for the two halves to reanneal and complete the repair without the loss of the sequence at the site of the break.

HR starts with the resection of 5′ ends by the MRN complex (MRE11, RAD50 and NBS1) [15,16], assisted by several nucleases and regulated by multiple factors, including CtIP, BRCA1 and BARD1 [17,18,19,20,21,22,23,24,25]. CtIP activates MRE11 endonuclease activity [26]. MRE11 incises the 5′-terminated strand at up to several hundred nucleotides from the break and degrades the cleaved strand through its 3′-5′ exonuclease activity in a process called short-range resection [26,27]. The short-range resection is followed by long-range resection, which removes up to several kilobases of DNA due to the coordinated action of exonucleases (EXO1, DNA2) and helicases (BLM, WRN) [21,28,29,30]. The process is directly stimulated by BRCA1-BARD1 [17,18]. Another important function of the BRCA1-BARD1 complex is to prevent the loading of the c-NHEJ factor 53BP1 (see below), and thus commit the cell to HR [31,32]. The resection step generates a nucleoprotein filament consisting of 3′-tailed ssDNA coated with RAD51 recombinase [33]. Since naked ssDNA is rapidly bound by the single-strand binding protein RPA, mediator proteins are required to replace RPA with RAD51 [34]. BRCA2 is the key mediator protein in human cells promoting nucleoprotein filament assembly [35]. It is recruited to a DSB via its partner PALB2, which is, in turn, recruited to BRCA1 [36,37,38]. Next, the nucleoprotein filament catalyzes the homology search and strand invasion, resulting in a displacement loop (D-loop) in a process stimulated by RAD54, PALB2, BRCA1-BARD1 and RAD51AP1-UAF1 [39,40,41,42,43,44,45,46]. Although the two ends of the DSB are processed in a similar manner, only one forms the D-loop [47]. The D-loop is then extended by a DNA polymerase capable of performing displacement synthesis within the D-loop, likely POL δ, with possible contributions from other DNA polymerases [48]. Depending on the subsequent steps, HR can be divided into three subpathways: the double Holliday junction pathway (dHJ), break-induced replication (BIR) and synthesis-dependent strand annealing (SDSA) [10]. SDSA is the predominant mechanism in mitotic cells. In this pathway, the D-loop disintegrates quickly and the extended 3′ end anneals to the non-extended 3′ end on the other side of the break [49,50]. The dHJ pathway occurs when both 3′ ends pair with a homologous sequence, prime DNA synthesis and are ligated to form classic dHJ structures that can be resolved into crossover or non-crossover products [51]. BIR happens when one of the two parts of the broken chromosome is inaccessible., and D-loop formation is followed by replisome assembly and replication through the rest of the chromosome [52,53].

In most cases, a sister chromatid is used as a donor in HR and the pathway is considered error-free compared to other DSBR pathways [54]. Nevertheless, HR can also be mutagenic to some extent due to possible genome rearrangements and small-scale mutations. Genome rearrangements arise from erroneous donor choice, while the increased rate of polymerase errors and the intrinsic chemical instability of ssDNA may lead to small-scale mutations [48,55].

### 2.3. Canonical Non-Homologous End Joining (c-NHEJ)

c-NHEJ is a predominant DSBR pathway in human cells (Figure 1B). Fundamentally, it centers around the re-ligation of broken DNA. Ligatable DNA ends can be directly processed by core c-NHEJ factors KU70/80, DNA-PKcs, XRCC4, XLF and DNA ligase 4 (LIG4) in a process starting within seconds after DSB formation [56,57,58]. The KU70/80 heterodimer is a DSB sensor that tightly encircles DNA and serves as a recruitment point for downstream c-NHEJ effectors [59]. Among these effectors is DNA-PKcs, a constituent of the phosphoinositide 3-kinase-related kinase family. DNA-PKcs, together with DNA-Ku complexes, forms the DNA-PK enzyme [60]. The kinase activity of DNA-PKcs is stimulated upon DNA binding, promoting the phosphorylation of various DNA repair proteins and the autophosphorylation of DNA-PKcs [61].

The ligation step requires precise alignment of DNA ends within a synaptic complex, comprised of two DNA ends, two KU70/80 heterodimers and two DNA-PKcs molecules. The synaptic complex adopts long-range and short-range conformations [62]. The long-range synaptic complex brings the two DNA ends into proximity, while, in the short-range synaptic complex, the DNA ends are aligned for ligation [63]. The kinase activity of DNA-PKcs, along with XRCC4-LIG4 and XLF factors, is required for the transition from the long-range to the short-range complex [62]. A single LIG4 molecule, stimulated by XRCC4,binds to both DNA termini and catalyzes the ligation of compatible ends immediately after the formation of the short-range complex,. This minimizes error-prone processing by other factors [64,65]. Interestingly, several studies show that LIG4 can tolerate certain terminal mismatches and damaged bases, a unique feature among vertebrate ligases [66,67]. 

DSBs that cannot be directly ligated may still be processed through a slow-kinetic subpathway of c-NHEJ, involving the activation of complex signaling pathways and the recruitment of multiple proteins [56,68,69]. The key player of slow-kinetic cNHEJ is 53BP1, also known as TP53BP1 [70]. 53BP1 recruits additional effectors, such as RIF1, SHIELDIN, CST and POL α/primase [31,71,72,73,74,75,76,77]. These proteins prevent the loading of HR factors and counteract the 5′ end resection, which is crucial for HR [31,71,72,73,74,75,76,77,78,79,80,81,82,83]. While 53BP1/RIF1/SHIELDIN/CST/POL α/primase largely counteract 5′ end resection, limited end trimming may still be necessary for c-NHEJ to generate ligatable ends. 53BP1 recruits PTIP [78], which, in turn, recruits ARTEMIS [84], a nuclease with both exo- and endonuclease activities [85]. The ARTEMIS nuclease activity requires the presence of KU70/80 and autophosphorylated DNA-PKcs [86]. ARTEMIS can remove both 3′ and 5′ overhangs, although only the processing of 5′ overhangs produces perfectly blunt ends [87]. DNA POL λ or POL μ carries out additional template-dependent or template-independent synthesis at 3′ termini to promote DNA-end synapsis and ligation [88]. 

### 2.4. Microhomology-Mediated End Joining (MMEJ or alt-EJ)

Although c-NHEJ is active in most cell types, early studies of c-NHEJ-deficient cells identified an alternative error-prone mechanism, often referred to as alt-EJ or microhomology-mediated end joining (MMEJ) (Figure 1C) [89,90]. Similarly to HR, MMEJ starts with the resection of 5′-terminated strands on both sides of the break, mediated by the MRN complex and CtIP, which is crucial for exposing microhomology-containing single-stranded regions [15,16,26]. Next, the two 3′ ends are aligned through the annealing of the exposed microhomologies, which can be as short as a few nucleotides (nt) in mammals [91]. If microhomologies are located at some distance from the break site, end bridging generates 3′ flaps that must be removed to complete the repair, followed by gap filling and ligation [89,90]. As a result, MMEJ is associated with deletions flanking the original DSB. The deleted region includes one of the two microhomologies and the region between them. Due to its mutagenic characteristics, MMEJ contributes to the plasticity of the genome but can also lead to chromosomal translocations, telomere fusions and carcinogenesis [16,89,90,92]. 

Poly ADP-ribose polymerase 1 (PARP1) and DNA polymerase theta (POL θ) are the key MMEJ factor [93,94,95]. PARP1 competes with KU for DNA end binding [96], promotes MRN recruitment [97] and facilitates end synapsis [98]. In addition, a recent study demonstrated the direct poly-(ADP)-ribosylation (PARylation) of POL θ by PARP1, which facilitates POL θ recruitment to DSBs [99].

POL θ is a multifunctional enzyme composed of an A-family DNA polymerase domain and an SF2 helicase-like domain separated by a large, unstructured central domain [100]. The helicase domain of POL θ promotes annealing [101], unwinds DNA with 3′-5′ polarity and facilitates strand displacement synthesis by the polymerase domain [102]. The low-fidelity polymerase domain fills in the gaps after the alignment of the 3′ ends and can accommodate various DNA structures including mismatched termini and ssDNA [95,103,104,105,106]. The 3′ flaps produced during MMEJ are removed by APEX2 and FEN1 [95,105,106]. The final ligation steps conclude the MMEJ repair pathway. In contrast to c-NHEJ, which relies on LIG4, LIG3 is the major contributor in MMEJ [98]. LIG3 forms a stable complex with the scaffold protein XRCC1, and both proteins interact with PARP1 [107,108].

### 2.5. Single-Strand Annealing (SSA)

Single-strand annealing (SSA) is the fourth DSBR pathway, discovered over 40 years ago but not studied as extensively as HR or c-NHEJ (Figure 1C) [109]. Mechanistically, SSA resembles MMEJ but relies on long direct repeats instead of microhomologies. It is usually associated with a higher degree of end resection and requires a different set of proteins [110,111]. SSA depends on resection factors MRE11, CtIP, EXO1 and DNA2 [112]. A characteristic feature of SSA is the annealing of 3′ ends by RAD52 in the presence of RPA [113,114]. In line with other studies, a recent cryo-EM structure [115] demonstrated that RAD52 forms a ring composed of ~10 subunits that binds ssDNA on the outer surface [113,116,117,118,119,120]. The structure also revealed a single RPA protein at the site of ring opening [115]. Based on observations that RPA stimulates SSA, excess RAD52 inhibits the reaction and there is no apparent interaction between RAD52 rings, the authors proposed a model of SSA in which RAD52 sporadically binds to RPA-coated ssDNA, replacing some of the RPA molecules on each of the two ends. Subsequently, the annealing of the two ends is promoted by the interaction between the loaded RAD52 rings on one strand and the remaining RPA on the other strand [115].

The annealing is followed by the removal of 3′ flaps by the ERCC1/XPF endonuclease [121,122,123]. The process concludes with gap filling and ligation, although it remains unclear which DNA polymerase and DNA ligase are involved [48].

### 2.6. DBSR Pathway Choice

c-NHEJ and HR are considered the major DSBR pathways. Their activity is regulated in many ways but is primarily controlled by the cell cycle [10,124]. In G1, c-NHEJ is a predominant pathway, whereas HR is largely considered inactive [125,126,127]. An exception to this rule is the demonstration of HR in G1 within highly repetitive ribosomal genes [128] and centromeric regions [129].

In S phase, both HR and c-NHEJ are active and contribute to the repair of two-ended DSBs [126,127]. At the same time, one-ended DSBs caused by replication fork blockage are repaired by HR but not by c-NHEJ [126,130]. In G2 phase, both c-NHEJ and HR are active [125]. In general, HR is considered to contribute less than c-NHEJ in G2. However, the exact proportion depends on the chromatin state and the complexity of the DNA ends induced by various damaging agents [131]. 

Repair kinetics studies suggest that c-NHEJ can be categorized into two types: fast-kinetic c-NHEJ, which is completed within 2–4 h after the break occurs, and slow-kinetic c-NHEJ, which operates over a timescale of approximately 24 h [125,131]. Fast-kinetic c-NHEJ is a predominant pathway in the G1 and G2 phases and accounts for the repair of ~70–80% of DSBs. The remaining DSBs are believed to be repaired by slow-kinetic c-NHEJ in G1 and by HR in G2, respectively [131,132]. Fast-kinetic c-NHEJ requires only the core c-NHEJ proteins, while slow-kinetic c-NHEJ is characterized by the recruitment of additional proteins, such as 53BP1 and ARTEMIS [133]. HR can be solely described by slow kinetics, operating on a timescale of ~24 h [125,134].

In the context of gene editing using SpCas9, repair half-life times ranging from 1.4 to 10.7 h have been demonstrated for different targets in K562 cells [135]. Interestingly, experimental data did not support a hypothesis of multiple rounds of SpCas9 cleavage and error-free repair until insertions or deletions (indels) are eventually generated. Instead, evidence supporting slow, error-prone repair was obtained [135]. This was an unexpected conclusion, given that SpCas9 produces blunt or nearly blunt ligatable ends and HR is active in K562 cells. A possible explanation for this is that SpCas9 remains bound to its target after cleavage [136,137]. This, in theory, may preclude fast-kinetic c-NHEJ and direct repair toward slow-kinetic c-NHEJ with the recruitment of additional factors that promote SpCas9 eviction and concomitant indels.

While c-NHEJ and HR can potentially result in error-free DSBR, SSA and MMEJ are inherently error-prone and must be tightly regulated. SSA is predominantly active during the S/G2 phase [122], consistent with its reliance on BRCA1, CtIP and the extensive end resection performed by HR proteins [110,112,138,139].

Mutations in BRCA1 that prevent its binding to PALB2 promote SSA [140]. SSA is also increased when RAD51 or BRCA2 is impaired [138]. Altogether, these findings suggest that SSA regulation throughout the cell cycle is similar to HR, but SSA is typically suppressed by the formation of the RAD51 filament.

MMEJ typically requires less extensive resection compared to SSA and HR, but it still relies on MRN and CtIP [110,141]. However, MMEJ is independent of BRCA1 and long-range resection factors BLM and EXO1 [25,141], meaning that unlike SSA, the decision between HR and MMEJ occurs during the resection stage. Experiments with synchronized cells have shown that MMEJ activity is lowest in G0/G1, gradually increases throughout the cell cycle and peaks in cells arrested in early mitosis [141]. In fact, MMEJ becomes the dominant DSBR pathway during mitosis when c-NHEJ and HR are repressed [142,143]. It is believed that MMEJ evolved to repair condensed chromosomes before cell division, thereby preventing genome instability at the cost of small deletions.

The network of DSBR pathways is highly complex, involving complicated signaling and chromatin remodeling that falls beyond the scope of this review. Additionally, there are multiple connections between different pathways, such as c-NHEJ proteins contributing to HR [125] and, vice versa, HR proteins contributing to c-NHEJ [133]. Numerous studies explore how to manipulate cells into selecting a specific DSBR pathway, a topic that will be discussed in the next chapter.

## 3. Inhibitors of DNA Repair Mechanisms in Gene Editing

### 3.1. HDR and Inhibitors of c-NHEJ and MMEJ in Gene Editing

**Figure 2:**
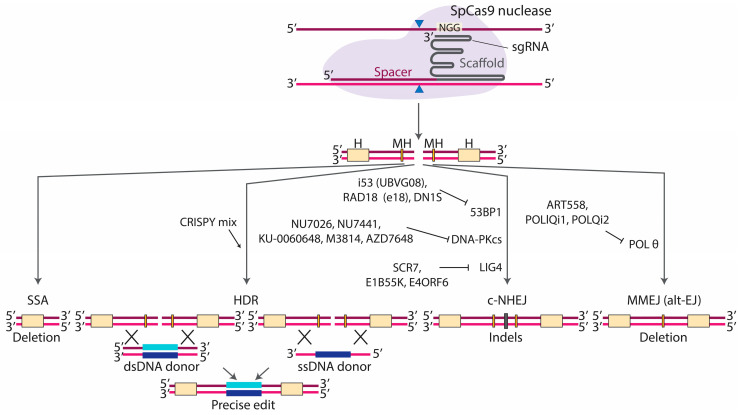
SpCas9-mediated targeted knock-in is installed via homology-directed repair (HDR) and is counteracted by competing repair pathways. SpCas9 paired with a single guide RNA (sgRNA) recognizes a DNA target and introduces a DSB. sgRNA includes a spacer and a scaffold. The target must be complementary to the spacer and must contain an adjacent Protospacer Adjacent Motif (PAM) sequence (NGG in the case of SpCas9). The resulting DSB can be repaired using an exogenous double-stranded or single-stranded donor via HDR to install the desired edit. Alternatively, c-NHEJ may result in generation of indels, MMEJ may lead to deletions between microhomologies (MH) and SSA may lead to deletions between larger homology sequences (H). Small-molecule or protein inhibitors of c-NHEJ or MMEJ proteins enhancing HDR are shown. The CRISPY mix consists of several components, including compounds with poorly characterized mechanisms of action. Thus, we present the CRISPY mix as promoting HDR without specifying its targets.

While Cas9 and sgRNA alone are enough for disrupting genes, an exogenous double-stranded or single-stranded donor DNA molecule is required for targeted insertions (Figure 2). Double-stranded DNA or single-stranded oligodeoxyribonucleotides (ssODNs) can be used, but the latter approach is more popular due to its higher efficiency [137,144,145]. HDR with dsDNA templates uses the RAD51-dependent HR pathway discussed in Chapter 2, while HDR with ssDNA is RAD51-independent and involves proteins from the Fanconi Anemia pathway through an unknown mechanism [137]. Both approaches can be compromised by c-NHEJ and MMEJ competing with HDR for the substrate and diluting the intended edit with undesired indels. Hence, many groups have been working on inhibiting c-NHEJ/MMEJ for efficient gene editing (Figure 2).

#### 3.1.1. Inhibitors of c-NHEJ

Given that c-NHEJ has the fastest kinetics in terms of repairing DNA in human cells [146], inhibiting c-NHEJ is of particular interest. c-NHEJ is initiated by the binding of the KU70/KU80 heterodimer to DNA. In 2016, Weterings et al. developed STL127705, the first compound to inhibit the interaction between KU70/KU80 and DNA, both in vitro and in vivo [147]. While STL127705 has been applied in cancer therapy [148], no studies to date have shown its effect on improving the efficiency of precise genome editing. Moreover, treatment with STL127685, a 4-fluorophenyl analog of STL127705, showed no improvement in Cas9 editing with an ssDNA donor [148].

In 2012, Srivastava et al. identified a compound called SCR7, which inhibited DNA LIG4 binding to DNA, leading to the accumulation of DNA breaks and the activation of intrinsic apoptotic pathways [149]. Maruyama et al. have successfully used SCR7 to promote HDR using dsDNA in epithelial cells (A549) and melanoma cells (MelJuSo) and ssODN in mouse embryos [150]. At least two other studies confirmed the positive effect of SCR7 on HDR [151,152]. However, other studies did not detect significant improvements in gene editing upon treatment with SCR7, questioning its potency, selectivity and utility as an HDR booster [153,154,155,156,157]. An alternative approach resulted in up to a seven-fold enhancement in HDR through the co-expression of adenovirus 4 E1B55K and E4ORF6 proteins, mediating the ubiquitination and proteasomal degradation of DNA LIG4 [151].

DNA-PKcs is probably the most promising c-NHEJ target, with multiple small-molecule inhibitors developed and tested in gene editing experiments, such as NU7026, NU7441, KU-0060648, M3814 and AZD7648 [157,158,159,160,161,162,163]. In 2013, Maresca et al. showed that a DNA-PK inhibitor, NU7026, increases the efficiency of HDR with zinc finger nucleases [164]. Other groups later demonstrated a 1.2–2.5-fold HDR improvement with NU7026 and Cas9 [146,153,165].

In 2015, Robert et al. demonstrated that NU7441 and KU-0060648 decrease c-NHEJ events with a concomitant ~2-fold increase in HDR in HEK293T cells edited with Cas9 [166]. In line with this, up to a 1.4-fold improvement in HDR upon NU7441 treatment was reported for iPSCs [146,167].

Another potent DNA-PKcs inhibitor is M3814 (also known as MSC2490484A, nedisertib or peposertib) [163,168]. Riesenberg et al. demonstrated that M3814 increases ssDNA-mediated HDR in hiPSCs, hESCs and K562 cells by ~2–10-fold depending on the cell line and the target [162,169]. In another study, M3814 caused a 3-fold increase in HDR with Cas9 and an AAV6 DNA donor [146].

In 2019, Fok et al. described AZD7648 as a potent and selective DNA-PKcs inhibitor that promotes tumor regression in combination with other agents that target the DNA damage response [161]. Our group demonstrated that AZD7648 led to a 2.9-fold improvement in HDR with an ssODN in HEK293T cells [157]. Recently, Matthew Porteus’s lab compared several DNA-PKcs inhibitors, including AZD7648 and M3814, and concluded that AZD7648 was more potent [170].

53BP1 is another target for c-NHEJ inhibition since it prevents end resection and BRCA1 recruitment [31,171]. To our knowledge, no small-molecule inhibitors of 53BP1 have been tested thus far while other approaches have been successfully applied to inhibit 53BP1. Since 53BP1 recognizes ubiquitylated histones at DSB sites, Canny et al. suggested that ubiquitin variants can be used as 53BP1 inhibitors. Indeed, several ubiquitin variants binding to 53BP1 were found. One of them, UBVG08 (also called i53), selectively binds 53BP1 in cells, inhibiting its accumulation at DSBs while promoting BRCA1 recruitment and HDR with dsDNA and ssODN donors [172]. The expression of an engineered variant of RAD18 (e18) is another way to improve HDR, since this protein competes with 53BP1 for binding ubiquitylated histones [173]. Fusing Cas9 to a dominant negative variant of 53BP1 (DN1S) increases HDR by reducing recruitment of 53BP1 specifically to Cas9-induced breaks without globally affecting the c-NHEJ pathway [174]

#### 3.1.2. Inhibitors of MMEJ

The repertoire of developed inhibitors of MMEJ is notably smaller compared to that of c-NHEJ, with DNA polymerase θ (encoded by the *POLQ* gene) being the primary target. Several studies have indicated that knockout or knockdown of *POLQ* partially decreases MMEJ-associated deletions and reduces the unwanted on-target effects of Cas9, such as translocations or large deletions [91,94]. However, only a limited number of small-molecule inhibitors targeting POL θ have been documented to date.

In 2021, Lord et al. presented ART558, targeting the polymerase function of POL θ. In their study, they demonstrated its efficacy in inhibiting the principal POL-θ-mediated DNA repair pathway, theta end joining [175]. Furthermore, in 2022, Heald et al. validated the effects of ART558, showing that it replicates the phenotype of POL θ loss [176]. Specifically in the context of gene editing, a 2023 study reported that this inhibitor prevents the formation of large deletions and facilitates HDR at a *GFP* gene stably integrated into the genome of mESCs [177]. However, no significant HDR enhancement was revealed for another target in mESCs and several human cell lines [177]. Similarly, the results from our lab demonstrate that a single *POLQ* knockout does not influence HDR efficiency in HEK293T cells [157].

#### 3.1.3. Combination of Inhibitors

While the inactivation of POL θ alone only has a marginal effect or no effect on HDR, dual inhibition of c-NHEJ with NU7441 and MMEJ with ART558 leads to a consistent improvement across multiple cell lines [177]. Our research group tested POLlQi1 (WO2021/028643) and POLQi2 (WO202/0243459) inhibitors in combination with the DNA-PKcs inhibitor AZD7648 [157]. POLQi1 targets the polymerase domain of POL θ and POlQi2 inhibits its helicase activity. AZD7648 in combination with POLQi1 or POLQi2 results in a consistent increase in HDR rate across various cell lines and targets, outperforming treatment with AZD7648 alone. The combined treatment, termed 2iHDR, not only enhances templated insertion efficiency but also greatly decreases indels and Cas9 off-target effects [157]. Riesenberg et al. came to a similar conclusion using the DNA-PKcs inhibitor M3814 and *POLQ* siRNAs, a combination called “HDRobust” [169].

Simultaneous inhibition of c-NHEJ and MMEJ is not the only available strategy. Riesenberg and Maricic tested various small-molecule inhibitors and found a combination, termed the “CRISPY” mix, which improves targeted insertions with ssODNs [153]. In addition to NU7026, discussed above, the mix includes trichostatin A, MLN4924 and NSC 15520. Trichostatin A is a histone deacetylase inhibitor that activates an ATM-dependent DNA damage signaling pathway [178]. MLN4924 promotes HDR by inhibiting the neddylation of unknown CtIP-interacting proteins and consequently promoting DNA end resection [179]. NSC15520 (fumaropimaric acid) blocks the binding of RPA to p53 and RAD9, possibly increasing the abundance of available RPA [180,181]. The “CRISPY” mix promoted HDR in hiPSCs and hESCs. However, in non-pluripotent cell types, some of the “CRISPY” components had an opposite effect, highlighting the difference in DNA repair in different cell lines [153].

## 4. Evolution of Prime Editing

### 4.1. At the Origin of Double-Strand-Break-Free Editing Methods

DSBs impose a risk of inversions, translocations, chromotripsis or long deletions spanning several kilobases [182,183,184]. To avoid DSBs, David Liu’s group developed a base editing approach. Base editing relies on a catalytically-inactive Cas9 nuclease (dCas9) fused to a cytidine or adenosine deaminase [185,186]. The binding of dCas9-sgRNA to the target results in an R-loop, in which ssDNA is accessible to the deaminase, eventually leading to C→T or A→G substitutions. Although this technology is a safer alternative to a classic HDR-based approach initiated by a DSB, the application of base editing is limited only to transition mutations; no insertions or deletions can be installed using this method.

**Figure 3:**
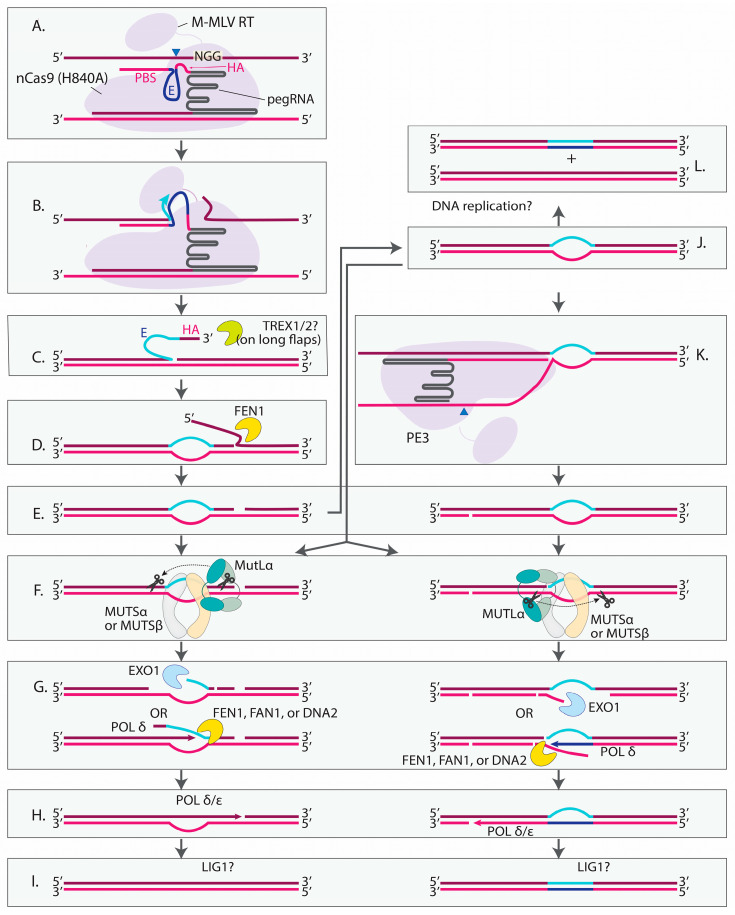
Prime editing (PE) and its interaction with mismatch repair (MMR) and other factors. (**A**) A typical prime editor consisting of an SpCas9 H840A nickase (nCas9) and a Moloney Murine Leukemia Virus (M-MLV) reverse transcriptase (RT) cleaves the nontarget strand of DNA (the strand that is not complementary to the spacer as opposed to the target strand annealed to the spacer). The **PE** guide RNA (pegRNA) includes a spacer, scaffold, homology arm (HA), the desired edit (E) and a primer-binding site (PBS). (**B**) The PBS binds to the cleaved nontarget strand. RT extends the DNA 3′ end using the edit and homology arm RNA sequence as a template. (**C**) The generated 3′ flap is likely subjected to degradation by cellular nucleases. TREX1/2 is a possible candidate that may act on long insertions. (**D**) The 3′ flap containing the edit hybridizes to the PAM-proximal side of the nick, replacing the initial sequence. The displaced 5′ flap containing the initial sequence is removed by the FEN1 endonuclease. (**E**) Following the removal of the 5′ flap, an intermediate is formed which contains a mismatch and single-strand break. (**F**) Mismatches of up to 13 nt introduced by the prime editor are recognized by either the MUTSα or MUTSβ complex, which recruit MUTLα. MUTLα cuts the strand containing a pre-existing break. In the context of **PE**, there is a higher probability of cleaving the edited strand due to the nick introduced by nCas9 (**F**, **left**). MUTLα cuts 5′ and 3′ to the mismatch. (**G**) The part of the incised strand which contains the mismatch is removed due to EXO1 5′-3′ exonuclease activity. Alternatively, POL δ synthesizes DNA while simultaneously displacing the incised strand without EXO1 involvement. In this case, the generated 5′ flap is removed by FEN1, FAN1 or DNA2. (**H**) The remaining gap is filled by a DNA polymerase, likely POL δ or POL ε. (**I**) The nicks are ligated, presumably by LIG1. As a result, either the initial sequence (**I**, **left**) or the desired edit is installed (**I**, **right**). (**J**) In the case where the nCas9-introduced nick is ligated prior to MMR, the subsequent MUTLα cleavage may happen either in the edit-containing strand (**F**, **left**) or the strand without the edit (**F**, **right**), depending on which strand contains pre-existing single-strand breaks generated independently from **PE**. (**K**) When the PE3 system is used, a second guide RNA without a pegRNA extension directs nCas9-RT to cleave the unedited DNA strand, thus promoting MMR to act on the unedited strand (**F**, **right**). This eventually leads to the installation of the desired edit (**I**, **right**). The nicking guide can direct Cas9-RT to cleave 5′ or 3′ of the mismatch (here, only cleavage of 3′ of the mismatch is depicted). The precise order of events has not been studied; PE3 cleavage may also occur **earlier**. (**L**) Given that **PE** is observed even when MMR is inhibited and since MMR is inactive on long contiguous mismatches, there should be an alternative way of resolving **PE** intermediates. Our hypothesis is that DNA replication may serve as this resolution mechanism in dividing cells, although other mechanisms may exist.

In pursuit of a technique to install various types of edits without DSBs, the same group developed the revolutionary PE technology, which can be used to install any types of base substitutions, small deletions or insertions, greatly expanding the genome editing toolbox (Figure 3) [187]. PE relies on a prime editing guide RNA (pegRNA) and a Cas9 nickase fused to a reverse transcriptase (RT) (Figure 3A). Similarly to a regular guide RNA, the pegRNA is comprised of a spacer on the 5′ end and a scaffold sequence but also contains a unique 3′-terminal extension. This extension includes the sequence complementary to the region upstream of the cleavage site (primer-binding site, PBS), the desired edit, and the sequence homologous to the region downstream of the cleavage site (the homology arm, HA). Once Cas9 introduces a nick to a DNA strand displaced after the spacer annealing, the PBS hybridizes to the PAM-distal part of the nicked strand. The RT reverse transcribes the desired edit and HA into the genome using the 3′ end of the nicked strand as a primer (Figure 3B). This results in a single-stranded 3′ flap (Figure 3C), which is able to hybridize to the PAM-proximal side of the nick, generating a 5′ flap that does not contain the edit (Figure 3D). The 5′ flap removal, nick sealing and the processing of the resulting heteroduplex by DNA repair proteins lead to a stably incorporated genomic edit (Figure 3E–L).

In the years following the first publication on PE, many research groups have been working on optimizing different components of the system (including the guide RNA, Cas9 and RT), developing new approaches inspired by the original PE concept, or manipulating cellular factors to improve the efficiency and favor the desired editing outcome. In the next part of the review, we summarize major achievements in the field with a focus on the mechanisms of DNA repair and possible ways to control this process.

### 4.2. From PE1 to PE7 and Beyond

Seven generations of SpCas9-based prime editors have been developed (summarized in Table 1). The first-generation prime editor (PE1) was composed of the wild-type M-MLV RT fused to the C-terminus of SpCas9 (H840A) nickase [187]. This approach enabled 0.7–5.5% editing efficiency for transversion mutations, ~4% for a small deletion and ~10–17% for short insertions. The second generation (PE2) introduced five amino acid substitutions into M-MLV RT, improving its in vitro substrate binding, processivity and thermostability [188,189]. This led to a ~5-fold increase in editing efficiency compared to PE1 [187]. PE3, PE4 and PE5 further enhanced PE by reducing the inhibitory effect of mismatch repair through the addition of a nicking guide or a dominant negative variant of the MLH1 protein (see more details below) [187,190]. Protein optimization using phage-assisted continuous (PACE) or non-continuous (PANCE) evolution combined with rational engineering led to the development of PE6, further subdivided into PE6a-g [191]. RT mutants PE6a-c were optimized for a smaller editor size, while PE6c-d demonstrated an increased activity on long, highly-structured templates. In addition, several SpCas9 variants (PE6e-g) with enhanced activity on a subset of targets appeared in the course of the evolution (PE6e-g [192]. Finally, PE7 was developed, which is a prime editor fused to the RNA-binding domain of the LA protein [191]. The LA protein binds to U tracts of the 3′ ends of RNA polymerase III transcripts and stabilizes 3′ ends of polyuridylated pegRNAs in the context of PE [191].

It is worth noting that there are two major drawbacks of the current PE naming system. The first is a lack of a standard nomenclature for PE systems composed of several elements from different PE generations. For example, any PE6a-d RT variants can be combined with any PE6e-f Cas9 variants and may include a nicking guide (as in PE3 systems) and MLH1dn (as in PE4/5), but there is no consensus on how the resulting editor should be named. Second, many reported modifications have been made to PE systems to address challenges and increase editing efficiency, but these have not been formally classified as new PE generations.

One such challenge is the guide stability. Since the 3′ extension of the pegRNA is not protected by Cas9, it is prone to degradation by RNA exonucleases. The PE7 approach likely solves this problem by stabilizing the polyuridylated 3′ ends of pegRNAs [191]. But other approaches have been suggested as well, such as adding structured RNA motifs to 3′ ends [193,194,195], using untethered circular RT templates [196,197] or using exonuclease-resistant synthetic guides [198].

Recruiting helper proteins to the editor through aptamers, the Suntag system, or covalently fused linkers (as in PE7) is a common approach used to influence editing efficiency. The pioneer transcription factor P65 [199], chromatin-modulating peptides [200], RAD51 DNA-binding domain [201] and T5 5′ exonuclease [202] are examples of helper proteins integrated into PE systems to promote chromatin accessibility, stabilize ssDNA and remove 5′ flaps, respectively. Two helper peptides (NFATC2IPP1 and IGF1PM1) that boost the translation efficiency of the PE2 editor were discovered because of extensive screening [203].

Several groups have focused on modifying the SpCas9-RT sequence to improve the protein architecture, optimize Nuclear Localization Signals (NLSs) and RT codon usage, remove potential splice sites and introduce mutations to increase nuclease activity. These efforts resulted in more efficient prime editors, such as PE* [204], PEmax [190] and iPE-C/iPE-N [202]. Among them, PEmax is a widely used variant (Table 1), which includes SpCas9 with additional R221K and N394K substitutions that improve nuclease activity, a 34-aa linker containing a bipartite SV40 NLS, human codon-optimized RT and an additional C-terminal c-Myc NLS [190].

The proximity of a PAM to the target is a huge challenge for CRISPR-based editing (Figure 2). The PAM of the most widely used SpCas9, NGG, occurs only once in every 16 genomic loci. To broaden the set of editable targets, a near-PAM-less SpRY SpCas9 variant and SpCas9 variants recognizing NGA, NGCC and NG were engineered and tested in combination with PE [205,206,207,208,209,210]. In addition, orthologous Cas9 variants from Staphylococcus aureus (SaCas9, NNGRRT PAM, SaCas9KKH, NNNRRT PAM) and Francisella novicida (FnCas9, NGG PAM, RHA-FnCas9, YG PAM) have been tested. The FnCas9 variants cleave 6–8 nt upstream of the PAM, in contrast to SpCas9, which cleaves 3 nt upstream of the PAM. On average, they demonstrated lower editing efficiencies compared to SpCas9 [204,210,211]. Recently, Cas12a has been developed, which is the first prime editor based on the type V CRISPR-Cas effector protein. Cas12a demonstrated up to 40% editing efficiency in vitro [212].

Several groups have tried to substitute M-MLV RT with RTs from other organisms, however, so far, none of the natural RT proteins has surpassed the efficiency of M-MLV RT. The most successful attempts so far include the evolution of *Escherichia coli* Ec48 retron-derived RT and *Schizosaccharomyces pombe* Tf1 retrotransposon-derived RT, which have led to the development of PE6a, PE6b and PE6c (Table 1) [209].

Another promising approach is to replace RT with a DNA-dependent DNA polymerase. A fundamental difference from classic PE in this case is the requirement of a DNA template. Three research groups have tested this concept in different configurations. Liu et al. previously developed a split PE approach with untethered SpCas9 nickase and RT, but, in their recent work, they substituted RT with a replicative polymerase from *Bacillus subtilis* phage phi29 [196,213]. In this system, the phi29 DNA polymerase is fused to the MS2 coat protein (MCP) and tethered via the MS2 stem–loop to the template, which is separated from the guide RNA. Ferreira da Silva et al. developed an approach called click editing (CE) [214]. The CE editor is a fusion of an SpCas9 nickase, a 3′-5′ exonuclease-deficient Klenow fragment from *E. coli* DNA polymerase I, and an HUH endonuclease (HUHe) from porcine circovirus 2 (PCV2). The PCV2 protein forms a covalent phosphotyrosine adduct with the HUHe recognition sequence in the click DNA (clkDNA) template. Similarly to the approach of Liu et al., the DNA polymerase template is not fused to the guide in this case. Finally, the recent pre-print by Nguyen et al. describes a two-component chimeric oligonucleotide-directed editing (CODE) system with an architecture similar to the original PE design [215]. The CODE system includes a thermophilic Bst DNA polymerase from *Geobacillus stearothermophilus* fused to the SpCas9 nickase and a long chimeric cpegRNA with a DNA extension on the 3′ end composed of a PBS and the DNA polymerase template. These DNA-polymerase-based technologies will likely expand the toolbox of editors with new capabilities in the future.

While rational design and phage-assisted protein evolution have accelerated progress in PE optimization, AI-assisted in silico protein optimization is undoubtedly the next step. In a recent pioneering study, Jiang et al. combined protein language models with a top-layer regression model to perform several rounds of PE2 evolution [216]. In the first round, random mutants were selected and tested experimentally. In subsequent cycles, the model actively learnt from the data and predicted variants with improved efficiency for experimental validation. In seven rounds, with just 12 mutants tested in each, the model predicted several variants, with a ~1.5-fold improvement in editing relative to PE2. Surprisingly, most of the substitutions were in the C-terminal RNase H domain and were not uncovered by previous in vitro optimization methods, demonstrating that there is still room for improvement even for the relatively well-optimized PE2 editor.

### 4.3. Bidirectional Prime Editing Systems (Bi-PE)

**Figure 4:**
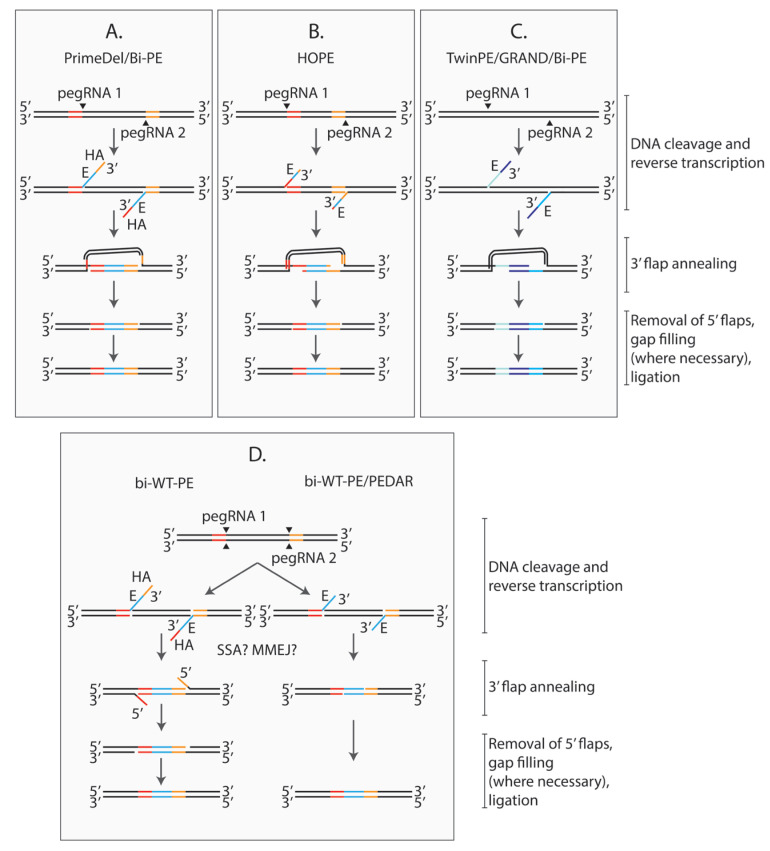
Bidirectional prime editing (Bi-PE) systems tested in human cells. (**A**–**C**) Nickase-based **Bi-PE** systems. Several methods are presented, all sharing the same principle: a nickase-based prime editor targets opposite strands of the same chromosome using a pair of pegRNAs. The pegRNA extensions are designed so that the resulting 3′ flaps are at least partially complementary to each other. The exact mechanism following the annealing of the 3’ flaps is unknown. The suggested process includes removal of 5’ flaps, gap filling, and ligation. Based on the composition of the 3’ flaps, **Bi-PE** approaches can be roughly divided into three categories: (**A**) In PrimeDel and a variation of Bi-PE, the 3’ flaps contain an edit (E) and a homology arm (HA). The HA of a flap connected to cleavage site 1 (pegRNA 1) is complementary to the region next to cleavage site 2 (pegRNA 2) and vice versa. Thus, in this case, the 3’ terminal part of each flap (the homology arm, HA) invades the homologous duplex while the remaining portions of both flaps hybridize to each other. This approach leads to the installation of edits accompanied by a deletion of the entire region between the two cleavage sites. Note that the edit may be removed from the flaps, resulting in clean deletions. (**B**) In the HOPE system, the 3’ flaps also share homology with the chromosome, but the homologous sequence directly follows the same nick site (black arrowheads). With this approach, a part of the region between the two nicks may be replaced with another sequence. (**C**) In TwinPE, GRAND and another variation of the Bi-PE approach, the 3’ flaps do not contain endogenous sequences. This approach allows one to replace the entire region between the two nicks with an exogenous sequence. (**D**) Nuclease-based **Bi-PE** systems. The method called PEDAR (Precise and Specific Deletion and Repair) or Bidirectional Wild-Type Prime Editing (Bi-WT-PE) involves a nuclease-based prime editor together with a pair of pegRNAs targeting opposite strands of the same chromosome. **The** cleavage (black arrowheads) splits the chromosome into three parts: the central part enclosed between the two cleavage sites (lost during subsequent repair) and two parts with 3’ flaps generated by prime editing. The two pegRNAs are designed with complementary edits. In addition, a HA may be present in the bi-WT-PE approach, similarly to the approach presented in A. The annealing of the two 3’ flaps generated by the nuclease-based systems may be mediated by SSA or MMEJ, but the precise mechanism is unknown.

PE successfully installs insertions of up to ~40 bp and deletions of up to ~80 bp [187]. To enable larger modifications, several groups independently tested TwinPE [217], GRAND [218], PRIME-Del [219], HOPE [220] and Bi directional PE (Bi-PE) systems [221] in human cell lines (Figure 4A–C). The five approaches share the idea of using two pegRNAs that target opposite DNA strands, encoding complementary or partially-complementary 3′ flaps that hybridize to each other. Deletions of ~1 kbp were generated using these methods with up to 30–80% efficiencies reported by different groups [217,221]. Choi et al. were able to obtain 10 kbp deletions at the *HPRT1* locus using PRIME-Del with ~0.8% efficiency measured by ddPCR [219]. Remarkably, sequencing amplicons with the deletion revealed that only ~3% of them contained additional indels [219]. Similarly, TwinPE demonstrated precise deletions of up to 780 nucleotides, including exon deletions with efficiencies up to 28% and only 5.1% indels [217]. These results demonstrate the high precision of deletions introduced by Bi-PE systems, as shown by several independent groups [217,221].

In addition to targeted deletions, dual-pegRNA strategies can be used to replace the target locus with a heterologous sequence [217,219,220,221]. Although such strategies inherit the inability of PE to integrate very long sequences, ~150 bp fragments were inserted with up to 63% efficiency due to splitting the sequence between the two partially overlapping flaps [218].

An alternative strategy for targeted insertions called template-jumping PE (TJ-PE) was suggested by Zheng et al., inspired by retrotransposon replication [222]. This method starts as regular PE, but the reverse-transcribed sequence includes a region at its 3’ end that serves as a PBS for a second reverse transcription reaction. A second guide RNA is used to introduce a nick in the opposite DNA strand at some distance from the initial nick. The nicked DNA 3′ end hybridizes to the initial 3′ flap, and RT initiates the second reverse transcription reaction. Using TJ-PE, 200 bp sequences were accurately integrated with ~34% efficiency and ~2% integration of 800 bp *EGFP* fragments was observed.

Overall, these results demonstrate the great potential of Bi-PE systems for skipping or rewriting exons, inserting minigenes and correcting complex genetic rearrangements.

### 4.4. Recombinase-Based Prime Editing Systems

Several PE proteins capable of making precise insertions and deletions in human cells have been described. However, these proteins currently cannot mediate insertions or deletions of sizes typical of exons or entire gene coding sequences. Such large DNA changes require extended pegRNA reverse transcription templates and long-range DNA polymerization, which significantly reduce editing efficiency. To address this limitation, several research groups have focused on recombinase-based prime editors, which use site-specific recombinases (SSRs). SSRs are enzymes that can excise, invert and integrate large DNA sequences in mammalian cells by catalyzing recombination between attachment sites *attB* and *attP* [223]. For example, one of the most used recombinases is Bxb1, a 500-amino-acid protein that binds *attP* and *attB* recognition sites that are 39 and 35 bp, respectively [224]. When PE is used to install one of the two attachment sites (e.g., *attB*) at a genomic location, a larger cargo can be subsequently integrated inside the *attB* sequence by Bxb1 if a donor DNA containing the *attP* site is provided.

This Prime-Editing-Assisted Site-Specific Integrase Gene Editing technology (PASSIGE) was introduced together with TwinPE, mentioned above [217,225]. At the time of initial publication, PASSIGE enabled highly efficient integration of the attachment site into the genome with more than 50% efficiency. When a clonal *attB*-containing cell line was isolated, subsequent transfection with Bxb1 and a donor plasmid led to up to 17% integration efficiency, demonstrating the huge potential of this technology. Yet a low efficiency when all reagents were delivered in a single transfection step (up to ~6.8%) remained a limitation [217]. In 2024, PASSIGE was significantly improved through the evolution of Bxb1 using PACE and PANCE. The evolved and engineered variants, evoBxb1 and eeBxb1 respectively, achieved 2.7-fold and 4.2-fold average improvements in the efficiency of targeted DNA integration, respectively. 

These improvements resulted in evolved PASSIGE (evoPASSIGE) and engineered PASSIGE (eePASSIGE). These systems achieved integration efficiencies ranging from 20% to 46% for multi-kilobase gene-sized cargo at commonly used and therapeutic loci after a single transfection [225].

In 2023, Gootenberg and colleagues introduced PASTE (Programmable Addition via Site-Specific Targeting Elements). In contrast to PASSIGE, which relies on TwinPE, PASTE relies on a classic PE approach with a single pegRNA. In addition, a serine integrase is directly fused to PE, rather than provided in trans. PASTE achieved the integration of DNA cargo of up to 36 kb in a single delivery reaction, with efficiencies of 50–60% in cell lines and 4–5% in primary human hepatocytes and T cells [226].

Interestingly, recombinase-based methods can also be used to correct genomic rearrangements. For example, in a model of Hunter Syndrome, multiplex TwinPE insertion of *attB* and *attP* into the genome, combined with Bxb1 recombinase, facilitated a 40 kb inversion [217]. Meanwhile, two other studies reported dual-pegRNAPE systems capable of inducing large deletions in human cells and plants [219,227].

Future advances in PE technologies together with the discovery and directed evolution of new recombinases are expected to enhance the capabilities and applications of recombinase-based technologies in PE [228].

### 4.5. The Role of DNA Repair Genes in Prime Editing

#### 4.5.1. Mismatch Repair (MMR)

In human cells, short mismatches or insertion/deletion loops (IDLs) of up to 13 nt are recognized by either the MUTSα complex (MSH2/MSH6 heterodimer) or the MUTSβ complex (MSH2/MSH3 heterodimer), which have partially-overlapping binding specificities (Figure 3F) [229,230,231,232,233,234]. MUTSα predominantly binds to base mispairs and short insertion/deletion loops (IDLs) that are up to 3 nt long [230,232,233]. In comparison, MUTSβ is essential for recognizing longer IDLs (~10 nt). It binds some shorter IDLs, but does not bind mispairs [229,233].

MUTSα/MUTSβ bound to a lesion recruits the MUTL complex, the main form of which, MUTLα, is a heterodimer of MLH1 and PMS2 (Figure 3F [235,236]. In the presence of MUTSα/MUTSβ, RFC and PCNA, MUTLα cuts the strand containing a pre-existing break near the mismatch [237]. MUTLα makes incisions 5′ and 3′ to the mispair [237]. The cuts made to the 5′ of the mismatch are of particular importance, because they serve as an entry point for the exonuclease EXO1, which is activated by MUTSα and removes a mismatch-containing patch in the 5′-3′ direction (Figure 3G) [238,239]. The generated gap is filled by DNA polymerase δ or ε holoenzyme (Figure 3H) [240,241,242]. Alternatively, POL δ synthesizes DNA while simultaneously displacing the mismatch-containing strand without EXO1 involvement [238]. The generated 5′ flap is removed by a 5′ flap endonuclease, FEN1, FAN1 or DNA2 (Figure 3G [238,243,244]. The final ligation step is likely performed by DNA LIG1, based on its interaction with PCNA, involvement in replication and ability to complete MMR in vitro (Figure 3I) [245,246]. 

The requirement of a pre-existing break for the cleavage by MUTLα is fundamental in eukaryotic MMR. Since the main function of MMR is the correction of replication errors, discontinuities in the newly synthesized DNA direct the repair machinery towards the daughter strand to preserve the initial sequence. This mechanism poses a challenge in PE, where the strand with an RT-introduced edit also contains a Cas9-generated nick. This problem was identified by Anzalone et al., who developed PE3 in an attempt to trick MMR by introducing a second nick in the nonedited strand (Figure 3K) [187]. The second cut is generated by the same SpCas9(H840A)-RT protein coupled to an sgRNA targeting the region 5′ or 3′ from the edit. This strategy increased editing efficiency ~1.5–4.2-fold compared to PE2 at four out of five tested targets. The efficiency of PE varied between different secondary cut sites with no apparent correlation between the percentage of intended edits and the location of the secondary cut relative to the initial cut site. In addition, the percentage of indels varied greatly depending on the location of the secondary cut; some sgRNAs did not change the indel level relative to PE2, while the others led to more than a 20-fold increase [187]. Habib et al. compared the frequency of indels between cells treated with PE3 and a similar system where the pegRNA was substituted with an sgRNA targeting the same site [247]. The PE3 system led to ~10% indels at two tested sites, while the same system with two nicks but lacking a 3′ flap resulted in almost no indels. These observations suggest that indels in PE3 are not directly caused by two nicks but rather arise because of the processing of flaps in the presence of a second nick in the opposite strand [247].

To decrease the probability of DSB formation, a strategy called PE3b was proposed in the same study [187]. The sgRNA in PE3b is designed to target the established edit, but not the initial sequence. To eliminate the risk of DSBs, the 5′ flap must be removed, and the edited strand must be ligated for the sgRNA-mediated nick to occur. The PE3b approach indeed demonstrated decreased levels of indels compared to PE3, although, in some cases, the percentage of prime edits was higher with the PE3 approach [187]. These findings highlight the importance of screening various sgRNAs to achieve the optimal edit/indel ratio when using PE3.

Though it was expected that MMR counteracted the establishment of the edit at the time when the concept of PE was introduced by David Liu [187], it was not proven genetically until two years later when the same group published the results of a CRISPRi screen [190]. In this study, Chen et al. tested how knocking down each of 476 genes involved in DNA repair or associated processes affected the efficiency of PE2 or PE3. *MLH1*, *PMS2*, *MSH2* and *MSH6* knockdowns led to the highest increase in the intended base substitutions, corroborating the inhibitory effect of MMR on PE. The increase in the intended edit was higher for PE2 (up to a 5.8-fold increase) than for PE3 (up to a 2.5-fold increase), but, in the case of PE3, the inhibition of the MMR genes also had a positive effect on the purity of editing outcomes by reducing indels. Deletions outside of the region between the pegRNA and sgRNA cut sites were especially responsive to MMR inactivation, suggesting that a part of them is caused by MMR. *EXO1* inactivation improved editing efficiency to a lesser extent compared to MUTSα or MUTLα, which is in line with the existence of EXO1-dependent and EXO1-independent pathways of MMR (Figure 3G) [237,238,239].

Another study by da Silva et al. explored the effect of 32 gene knockouts on PE and confirmed the inhibitory effect of MMR [248]. The only marked differences were the absence of the *MSH6* effect, observed by Chen et al., and an increase in PE in the *ΔMSH3* background. This discrepancy can be explained by the different substrates used in the two studies. Chen et al. used a single-nucleotide mismatch, the substrate of MUTSα (MSH2/MSH6 heterodimer), while da Silva et al. used a 5 bp deletion loop recognized by MUTSβ (MSH2/MSH3 heterodimer) [190,248]. A recent study by Park et al. highlights these differences, demonstrating that base mispairs and 1 nt IDLs are enhanced in *ΔMSH6* knockout cells; 5–10 nt IDLs are enhanced in *ΔMSH3* mutants; 3 nt IDLs are stimulated by either of the two deletions; and 15–34 nt IDLs are not affected by *MSH2/MSH3/MSH6* [249]. In addition, an shRNA screen performed by Li et al. revealed an inhibitory effect of MMR components *PMS2* and *MLH1* on 6 nt insertions, though no consistent results were observed for other MMR factors [250]. Overall, these genetic studies unequivocally demonstrate the inhibitory effect of MMR on PE. These findings are further supported by microscopy, revealing colocalization of a prime editor with MLH1 or MSH2 [248].

To our knowledge, there is no available small-molecule inhibitor of MMR. However, several other approaches have been suggested to leverage the effect of MMR inactivation on PE. Chen et al. suggested that contiguous mismatches are not well recognized by MMR, and therefore increasing the size of the edit by introducing silent mutations should promote PE [190]. Indeed, PE2 works ~2.7-fold better on 3-5-base contiguous substitutions compared to shorter 1-2-base mutations [190].

If the intended outcome is an insertion or deletion, the inhibitory effect of MMR should decrease with the increase in the IDL length. IDLs of up to 12 nt are still repaired by MMR in vitro when treated with extracts of HeLa cells, although at a lower efficiency compared to shorter IDls, and IDLs longer than 16 nt are unlikely to be recognized by MMR [251]. In line with this, Koeppel et al. demonstrated that the fold change difference in PE efficiency between *wt* and *ΔMLH1* cells decreases exponentially with increasing insertion length. According to their model, a ~23–28-fold difference is expected for 1 nt insertions, which drops by 40–48% for every additional nucleotide, approaching 1 for insertions longer than 13 nt. However, increasing the indel size is not always possible when it comes to editing a therapeutically-relevant target. In addition, if the intended outcome is a long insertion, other factors, such as RT processivity or the presence of secondary structures in the reverse transcription template, may lower efficiency [252].

Transient inactivation of MMR components using siRNA is another strategy to enhance PE, but it requires pre-treatment with siRNA for 2–3 days [190]. Transient degradation of the MLH1 protein tagged with dTAG is another alternative [248]. However, this method is even more time-consuming since it requires introduction of the tag into the genome.

Chen et al. suggested co-expressing the dominant negative variants of *MMR* genes together with a prime editor to achieve transient MMR inactivation [190]. Among several tested engineered MLH1, PMS2, MSH2 and MSH6 proteins, the MLH1 Δ754–756 variant (referred to as MLH1dn) added in trans demonstrated the highest results. The combination of PE2 with MLH1dn was designated as PE4, and the combination of PE3 with MLH1dn was designated as PE5 (Table 1). Alternatively, an MLH1NTD–NLSSV40 variant, which is less efficient but smaller in size (355 aa vs. 753 aa for MLH1dn), can be used. Overall, the addition of MLH1dn improved the installation of all types of substitutions, although the effect was smaller for G-C to C-G edits. The G-C to C-G mutation is formed due to C-C mismatches that are not efficiently repaired by MMR and therefore have a higher basal editing level [190,253]. The efficiency of short 1 or 3 bp indel installation was also greatly improved by MLH1dn. However, the effect decreased with the size of the indel, and almost no difference between PE2 and PE4 was observed for indels ≥ 15 nt in length. The effect of MLH1dn on PE also depends on the cell line. The improvement is smaller in cells like HEK293T, in which MMR is already partially-inactivated, compared to MMR-proficient cell lines such as HeLa, K562 and U2OS [190]. These results demonstrate the great potential of PE4 and PE5 technologies for cell engineering and therapeutic application. However, there is also a possibility for a further improvement since the use of MLH1dn does not completely inhibit MMR, as evidenced by the higher activity of PE in *ΔMLH1* knockout cells.

#### 4.5.2. FEN1

Following the reverse transcription step, an intermediate with a 3′ flap, which can be converted into a 5′ flap, is formed (Figure 3C,D). The removal of the 5′ flap is a favorable process for PE, while the cleavage of the 3′ flap may lead to unintended editing outcomes. FEN1, a 5′ flap endonuclease that removes RNA primers of Okazaki fragments, is an obvious candidate for the role of the enzyme that removes 5′ flaps in PE [254]. Indeed, the results of the CRISPRi screen by Chen et al. revealed a decrease in PE2 and PE3 efficiency upon *FEN1* knockdown [190]. Li et al. also reported a significant decrease in PE in cells expressing *FEN1* shRNA [250]. The observed decrease was less than 2-fold in both studies, suggesting either functional redundancy with other proteins or knockdown that was not efficient.

#### 4.5.3. HLTF

DNA damage that hasve not been repaired prior to replication can stall the progression of a replication fork. To achieve the completion of replication despite the presence of damage, two DNA damage tolerance (DDT) pathways have evolved: translesion synthesis (TLS) and template switching (TS) [255,256]. The TLS pathway is initiated by the monoubiquitination of PCNA at K164 and relies on low-fidelity DNA polymerases replicating DNA across damaged bases [257]. The TS pathway is initiated by the polyubiquitination of PCNA at K164 [258,259,260]. It operates by switching the template from the damaged strand to the nascent daughter strand on the sister chromatid to bypass the damage [256,261].

The HLTF (Helicase-Like Transcription Factor) protein was initially characterized as a transcription factor binding to promoters and enhancers of various genes and hence its name he high degree of sequence similarity between HLTF and its closest ortholog, RAD5, which is central to the TS pathway in *Saccharomyces cerevisiae*, prompted studies of HLTF function in the DNA damage response and led to the discovery of its role in TS. Similarly to RAD5, HLTF is an E3 ubiquitin ligase (E3) and catalyzes PCNA polyubiquitination [260]. The DNA-binding domain of HTLF recognizes 3′ ends of stalled replication forks and the helicase domain promotes fork reversal [262,263]. Interestingly, a recent study demonstrated a direct interaction between HLTF and MSH2 in human cells [264]. However, the functional significance of this interaction for MMR or TS remains unknown.

Chen et al. demonstrated a weak stimulating effect of *HLTF* on PE2 and an inhibiting effect on PE3 in two different cell lines with a single-nucleotide substitution as the desired edit [190]. In contrast, Li et al. observed an upregulation of 6 nt insertions installed via PE2 upon knocking down *HLTF* [250]. This study also revealed that the inhibitory effect of *HLTF* on PE2 varies across genomic targets, with actively transcribed genes being more responsive to *HLTF* knockdown compared to non-transcribed regions. At the same time, only minimal changes in gene expression and chromatin accessibility were revealed in cells with *HLTF* knockdown, suggesting that its effect on PE is not related to its role as a transcription factor. It is yet to be determined whether HLTF influences PE through its function in TS, the interaction with MSH2, or another uncharacterized mechanism. The reason for the opposite effects of *HLTF* on PE2 vs. PE3, as well as different types of edits, also remains unknown.

#### 4.5.4. TREX1 and TREX2

TREX1 (DNase III) and TREX2 are 3′-5′ exonucleases that are active on ssDNA and dsDNA [265,266,267]. The preferable substrate for both enzymes is a partial DNA duplex with mispaired 3′ termini like the 3′ flap generated during PE [265]. TREX1’s main role is to degrade cytosolic DNA and prevent inappropriate immune responses through a cGAS-STING pathway of DNA sensing [268,269]. Accordingly, TREX1 is predominantly localized in the perinuclear space or endoplasmic reticulum [270,271]. However, several studies detected TREX1 in nuclei under certain circumstances. For example, when cytolytic T cells and NK cells release Granzyme A (GZMA) into target cells through an immunological synapse, TREX1 moves to the nucleus to enhance DNA degradation during the caspase-independent cell death pathway [272]. More importantly, in the context of gene editing, TREX1 translocates to the nucleus upon UV, γ irradiation or hydroxyurea treatment in growing mouse cells, suggesting that TREX1 may play a role in DNA repair or damage tolerance, although the mechanism remains unknown [273,274]. ~3-fold decrease in unintended deletions with the PE3 approach was detected upon knocking down *TREX1* [190]. Therefore, it is likely that TREX1 is present in the nucleus and ready to trim DNA ends when PE occurs.

TREX2 is a nuclear protein that participates in DDT, processing stalled replication forks and promoting mutations through its 3′-5′ exonuclease activity and ability to ubiquitinate PCNA at K164 [252,275,276]. TREX2’s impact on genome stability depends on genetic backgrounds, but, at least in some cases, TREX2 degrades unprotected 3′ ends. For example, in mouse embryonic stem cells expressing human mutant RAD51 K133A defective in filament assembly, TREX2 contributes to the nascent strand degradation after treatment with hydroxyurea [276].

Although both TREX1 and TREX2 may theoretically antagonize PE by degrading 3′ flaps, their role in PE is not clear. Koeppel et al. tested the effect of TREX1 or TREX2 overexpression on the efficiency of insertions installed by PE2 and found that both enzymes interfere with prime insertions in a length-dependent manner [252]. While only up to 3-fold differences were observed for 1 nt insertions, *TREX1/2* overexpression led to a 20-180-fold decrease in editing for longer 30 nt insertions. However, the effect of *TREX1/2*’s loss of function on short vs. long PE insertions has not been studied and therefore it is not clear whether the observed differences reflect what happens in cells with a normal expression level. No effect of *TREX1* knockdown on base substitutions installed by PE2 or PE3 was detected in the CRISPRi screen by Chen et al. [190]. Similarly, the shRNA screen by Li et al. did not detect statistically significant differences for 6 bp prime insertions between cells with or without *TREX1* shRNA [250] It is unknown whether TREX1/2 only interferes with long insertions, and further experiments are required to support or refute this.

#### 4.5.5. LIG1

The 3′ flap must be ligated for the successful completion of PE. If the resulting heteroduplex is resolved through MMR, the MMR outcomes should also be ligated to restore DNA integrity. Chen et al. demonstrated a decrease in PE2 and PE3 upon *LIG1* knockdown in HeLa and K562 cells [190]. In contrast, Li et al. did not observe an inhibitory effect of *LIG1* knockdown on PE2 in K562 cells [250]. Therefore, further experiments are required to elucidate the impact of DNA ligases on PE.

### 4.6. Prime Editing Nuclease (PEn) and Associated DNA Repair Pathways

**Figure 5:**
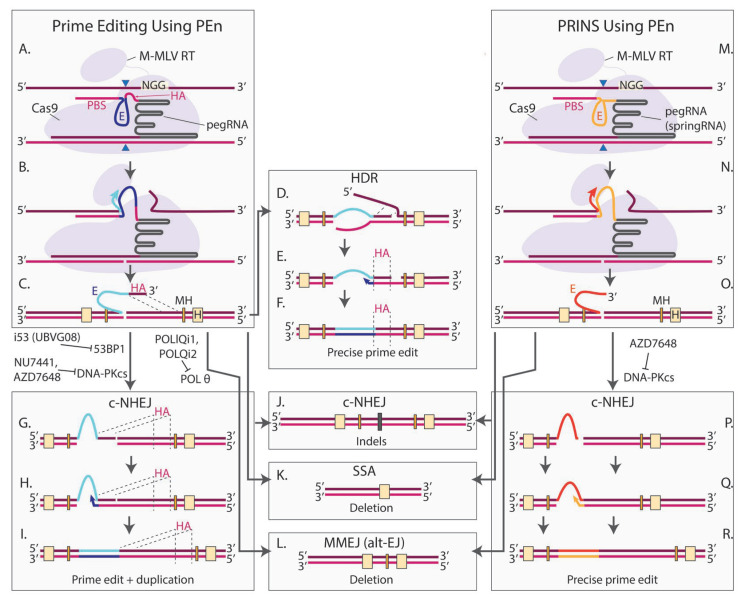
Nuclease-based prime editing(PE)technologies. (**A**–**C**) DNA cleavage and reverse transcription mediated by PEn (prime editing nuclease) and pegRNA. DNA cleavage and 3′ flap generation proceed similarly to the classic PE approach, except that a DSB is generated instead of a nick (**A**,**B**). Cleaved DNA with a 3′ flap containing a homology arm (HA, magenta) and an edit (E, light blue) is shown (**C**). Dashed lines denote the region in the chromosome that corresponds to the HA in the flap. This schematic depicts a scenario in which PEn substitutes a region downstream of the break with an edit. (**D**–**F**) A homology-directed mechanism leading to the correctly installed prime edit. While the exact mechanism is not known, it is governed by the HA in the flap, which needs to invade the homologous duplex at the other side of the break (**D**). In the case of a continuous substitution (depicted), the invasion will generate two flaps with the original sequence. The removal of the flaps, subsequent gap filling, and ligation (**E**) lead to successful installation of the edit (**F**). (**G**–**J**) Imprecise integration of the edit via c-NHEJ. In the case when strand invasion fails, c-NHEJ can align the two sides of the break, one of which contains the 3′ flap (**G**). Although the detailed mechanisms are not clear, subsequent gap filling and ligation (**H**) lead to the installation of the edit which is accompanied by the duplication of the HA (**I**). c-NHEJ may be accompanied by limited end resection, leading to indels (**J**). Inhibitors of DNA-PKcs and 53BP1 which have been shown to inhibit c-NHEJ following PEn editing are shown beside arrows that depict the sequential flow of events. (**K**) End resection may result in DSB repair via single-strand annealing (SSA), resulting in a deletion between two long homologous sequences denoted as ‘H’. (**L**) End resection may result in DSB repair via MMEJ, resulting in a deletion between two microhomologies denoted as ‘MH’. Inhibitors of POL θ which have been shown to inhibit MMEJ following PEn editing are shown beside arrows that depict the sequential flow of events. (**M**–**O**) DNA cleavage and reverse transcription mediated by PEnwith a pegRNA missing an HA (also called springRNA). The method is called PRimed INSertions (PRINS). DNA cleavage and 3′ flap generation proceed similarly to the stages depicted in A-C (**M**,**N**). Cleaved DNA with a 3′ flap containing only the edit (E, dark orange) is shown (**O**). Due to the absence of an HA, insertions are the only possible edit that can be installed. (**P**–**R**) Precise integration of the edit via c-NHEJ. c-NHEJ aligns the two sides of the break, one of which contains the 3′ flap (**P**), and ligates them (**Q**), although the detailed mechanisms are not clear. Gap filling and subsequent ligation lead to the installation of the intended insertion (**R**). Similarly to PE using PEn and pegRNA, processing of the break caused by PRINS editing may result in erroneous c-NHEJ (**J**), SSA (**K**) or MMEJ (**L**). The DNA-PKcs inhibitor, which has been shown to inhibit Choi PPRINS, is shown beside an arrow that depicts the sequential flow of events.

The efficiency of PE varies between cell lines. Adikusuma et al. replaced SpCas9 nickase with SpCas9 nuclease, as they hypothesized that the low efficiency of PE in HeLa and K562 cells might be due to inefficient 5′ flap resection and removal of the nonedited strand (Figure 5A). SpCas9 nuclease boosted the overall PE efficiency from 22% to 71% in K562 cells and from 6.7% to 30% in HeLa cells [277]. Motivated by the high efficiency in vitro, Adikusuma et al. next applied PEn to generate mice through zygote microinjection. Though the efficiency varied depending on the target and the edit type, remarkable results were achieved. For example, in some cases, up to 87.5% of the mice contained the intended edit with 3 nt insertions at the *CHD2* and *COL12A1* sites while 100% of the mice had the intended edit at the *TYR* site [277].

A few months after the first paper on PEn was released, three other research groups, including ours, published results on PE using SpCas9 nuclease [278,279,280]. Two of these studies demonstrated that, similarly to the TwinPE/Bi-PE/PRIME-Del/HOPE/GRAND technologies, PEn combined with a pair of pegRNAs targeting complementary strands can be used to delete large pieces of DNA and insert a short sequence encoded in the 3′ flaps (Figure 4D [279,280]. This method, called PE-Cas9-based deletion and repair (PEDAR)by Jiang et al. or bi-WT-PE by Tao et al., achieved ~3% deletion of a 16.8-megabase region in cell cultures, which is much larger than was previously reported for PRIME-Del [219,280]. In vivo, PEDAR enabled precise correction of a 1.38-kilobase pathogenic insertion disrupting the *FAH* gene in a mouse model of tyrosinemia [279]. In the presence of a tyrosine catabolic pathway inhibitor, ~1% of hepatocytes in PEDAR-treated mice expressed *FAH*. In the absence of the inhibitor, the corrected hepatocytes gained a growth advantage and repopulated the liver, leading to ~78% corrected alleles, demonstrating the potential therapeutic relevance of this method.

Tao et al. showed that this technology can also be used for installing inter-chromosomal translocations. Interestingly, most of the translocations were unbalanced, likely because the complementary 3′ flaps promote the joining of distant regions, while the respective PAM-proximal blunt ends remain non-ligated [280]. However, the exact mechanism is not known. The two complementary 3′ flaps are required for PEDAR, since the concomitant use of a pegRNA and an sgRNA fails to produce the correct edit [279]. Therefore, it is likely that the process is driven by MMEJ or SSA, but this awaits experimental validation.

More clarity on the DNA repair mechanism has been achieved for PEn with a single pegRNA. Adikusama et al. and later others noted that while PEn greatly improves the overall PE efficiency, many edits contain duplications of the homology arm sequence [277,278,280]. These imprecise edits might be products of DSB repair mediated by c-NHEJ when the 3′ flap is directly ligated to the PAM-proximal side of the break (Figure 5G–I), while the precise insertions could occur through a homology-dependent process (Figure 5D–F). Indeed, members of our group, demonstrated that the additional RT template integrations were abolished upon treatment with the selective inhibitor of DNA-PK, AZD7648 [161,278]. Moreover, at several loci, DNA-PK inhibition also led to an increase in total rates of correct insertions [278]. In 2023, Li et al. applied another DNA-PK inhibitor (NU7441) and demonstrated improved purity of the RT-dependent edits, reaching up to 75% precision, thus confirming the results obtained by our group [281]. They also tested the effect of inhibiting 53BP1, a protein promoting c-NHEJ by blocking the 5′ end resection required for HDR [281]. Previously it was shown that engineered ubiquitin variants prevent the recruitment of 53BP1 to DSBs [172]. Li et al. demonstrated that the UBVG08 variant, and especially its derivative G08(144A), also called i53, consistently promoted the levels of accurate edits by PEn at three tested endogenous sites [281]. This approach was called uPEn (ubiquitin-variant-assisted PEn). uPEn efficiently installed insertions (38%), deletions (43%) and substitutions (52%) in HEK293T cells [281]. Altogether, the results obtained by Peterka and Li demonstrate that inhibiting c-NHEJ is a promising approach to improve the precision of PEn.

While DNA-PK inhibition greatly reduced imprecise prime edits in experiments by Peterka et al., it did not change the percentage of unrelated indels. Strikingly, DNA-PK inhibition in *POLQ-/-* cells almost completely abolished indels without compromising precise editing [278]. At the same time, no difference in indels was observed between *wt* and *POLQ-/-* cells without DNA-PK inhibition. This important experiment demonstrates that c-NHEJ and MMEJ are redundant pathways responsible for the generation of byproducts, and a remarkable purity of prime edits can be achieved by the simultaneous inhibition of both pathways. Recently, Antoniou et al. tested this idea in *wt* cells treated with AZD7648 targeting DNA-PK and PolQi1/PolQi2 inhibiting POL θ [157,282]. Simultaneous treatment with all three compounds led to almost 100% purity in HEK293T and HeLa cells. This approach was called 2^+^iPEn. Similar precision was achieved with the nickase-based PE5 approach, but 2^+^iPEn’s overall efficiency greatly surpassed PE5 on some of the targets (although was lower on the others) [282].

Though homology arm duplications are very frequent in PEn without inhibitors, these events also occur in nickase-based PE approaches utilizing a second nicking guide. Adikusuma et al. demonstrated that such events were present in all tested PE3 target sites and comprised 5–40% of the unintended edits [277]. Therefore, it would be interesting to test if DNA-PK inhibitors further improve the purity of PE3 and PE5.

Once it became clear that PEn-mediated imprecise edits could be inserted through a homology-independent c-NHEJ mechanism, testing a pegRNA without a HA was the next step. The method was called PRINS (Single Primed INsertion) and the pegRNA without an HA was called springRNA (Single PRimed INsertion gRNA) (Figure 5M–R) [278]. Peterka et al. showed that PRINS can install an intended insertion with up to 50% efficiency across a panel of targets in various cell lines [278]. Data from an independent study also confirmed the ability of PRINS to install an insertion at three tested targets [280]. Precise PRINS editing was completely abrogated if cells were treated with the DNA-PK inhibitor, thus confirming that NHEJ is responsible for PRINS-mediated insertion [278]. We expect that PRINS will become a useful technology for editing cell lines with inefficient homology-based pathways. The limitation of PRINS is its inability to install modifications other than insertions.

## 5. Delivery of Prime Editing

Delivery strategies for PEcan be categorized into three main types: physical, chemical and viral. Physical delivery methods include microinjection and electroporation. Chemical delivery strategies involve liposomes and nanoparticles, while viral delivery commonly utilizes retroviruses, adenoviruses and adeno-associated viruses (AAVs), which are the most widely used vectors for delivering PE components [283]. In this chapter, we will focus on the delivery of PE using AAVs, lipid nanoparticles (LNPs) and virus-like particles (VLP).

AAV vectors are currently the leading platform not only for PE but also for various gene therapy applications. They can transduce both dividing and non-dividing cells and rarely integrate into the host genome [284]. Additionally, they are considered safe, as they are only mildly immunogenic [285]. However, the large size of the prime editor (~6.3 kb) exceeds the packaging capacity of AAVs (~4.7 kb) [286]. To overcome this limitation, several split PE systems compatible with dual-AAV vectors were developed, such as a system with untethered Cas9 and RT, several intein-split PE systems and a system with RT and Cas9 dimerized through coiled-coil peptides [210,287,288,289,290,291,292]. To further reduce the size of the editor, several groups tested RT mutants lacking the ΔRNaseH domain, which is dispensable for PE [210,289,291].

To achieve a single AAV delivery, a 4.5 kb mini-PE editor composed of compact *Campylobacter jejuni* Cas9 (CjCas9 H559A) and truncated M-MLV RT was developed [293]. However, it demonstrated only a maximum of 10% editing efficiency in vitro and less than 1% in vivo [293]. The application of the CjCas9 editor was also limited by a long N3VRYAC PAM [294,295]. Recently, a promising evoCjCas9 variant has been derived using PANCE and PACE [296]. This variant demonstrates higher PE efficiencies and supports editing at non-canonical PAMs. Though the evoCjCas9-RT prime editor has not been tested in vivo with an AAV yet, a single AAV delivery of an evoCjCas9 base editor led to 41% editing in hepatocytes and up to 34% in mouse brain [296]. Several new approaches have since targeted models for brain, eye and liver diseases [196,204,292,297]. A study published in 2024 reported that dual-AAV delivery achieved up to 42% editing efficiency in the brain cortex, 46% in the liver and 11% in the heart [287]. Another recent study demonstrated 17.5% editing efficiency with split-AAV9 delivery at the *PCSK9* gene, which is involved in cholesterol homeostasis [298]. To further enhance therapeutic relevance, size reduction of PE components could improve the dual-AAV system by simplifying usage and increasing efficiency.

To address the size limitations of viral delivery and improve safety, alternative methods such as lipid nanoparticles (LNPs) and virus-like particles (VLPs) have been developed. LNPs typically consist of four components: ionizable lipids, cholesterol, a helper lipid and a PEG–lipid conjugate. These components form uniform spheres capable of encapsulating RNA payloads. The component ratio significantly affects LNP activity, toxicity and transfection efficiency. LNPs can deliver PE components and other CRISPR tools, such as mRNA-Cas9 LNPs, which offer efficient loading, design flexibility, and biocompatibility, making them a key player in clinical-stage gene editing for CRISPR therapies [299]. In 2023, Chen et al. successfully delivered PE mRNA via LNPs in an immunodeficient mouse model [300]. Herrera-Barrer and colleagues achieved 54% PE efficiency in a reporter cell line using enhanced LNPs (eLNPs) [301]. Despite these advancements, LNP accumulation in the liver restricts their applicability to non-hepatic tissues. Strategies for targeting specific cells or organs are under development to address this issue [299].

Virus-like particles (VLPs) are non-infectious viral capsid/envelope structures that deliver gene editing agents such as mRNA or proteins, reducing the risk of viral genome integration and off-target effects. Currently, lenti/retrovirus-based VLPs are the most used VLPs for PE delivery [302]. While most studies focus on base editing, An et al. demonstrated in 2024 that subretinal injection of v3-PE-eVLPs achieved 15% editing efficiency in a mouse model of retinal degeneration [303]. Very recently, Nanoscribes, a new type of engineered VLP, achieved up to 25% editing efficiency in myoblasts, hiPSCs and hiPSC-derived hematopoietic stem cells [304]. Given the potential of VLPs in base editing, their application in PE is expected to expand significantly in the coming years.

## 6. Safety

### 6.1. Off-Target Effects

Evaluating the safety is a pivotal aspect when considering PE as a potential therapeutic tool. PE requires not only target–guide RNA complementarity, as with other Cas9-based methods, but also target DNA-pegRNA PBS complementarity to initiate pegRNA-templated reverse transcription and target DNA-RT product complementarity for flap resolution. Hence, researchers hypothesized that these two additional DNA hybridization steps could reduce off-target PE.

In 2019, Liu and colleagues showed that PE2 and PE3 induce much lower off-target editing than Cas9 at known Cas9 off-target sites [187]. With the gradual improvement of PE systems and their on-target editing efficiencies, off-target effects have been evaluated multiple times and remained consistently low. For example, PE4, PE6 and PE7 did not substantially increase off-target PE [190,191,192]. Engineered pegRNA (epegRNA) did not exhibit a higher level of off-target editing compared with pegRNAs [195]. At the same time, while nuclease-based prime editors such as PEn and uPEnshowed very high on-target editing efficiency they also promoted off-targets at a level comparable to the Cas9 nuclease alone, highlighting the need for stringent peg/springRNAs and high-fidelity Cas9 enzymes.

Recently, several new methods to assess the profile of PE off-target sites have been developed. In 2023, Wolfe and colleagues published PE-tag, an approach for the genome-wide identification of prime editor activity. In the same study, they show that off-target editing rates are influenced by pegRNA design [305]. In 2022, Chen and colleagues developed a platform to profile guide-independent off-target effects in human cells. Using this approach, they demonstrated that PE3 does not cause guide-independent off-target mutations in DNA or RNA, as well as alterations in telomeres, confirming the high specificity of its reverse transcriptase moiety. In the same year, Lee and colleagues published TAgmentation of Prime Editor sequencing (TAPE-seq), another method to determine off-target candidates for PE [306].

### 6.2. DNA Repair Considerations

While modulating DNA repair may be an efficient strategy to improve PE, associated perturbations potentially leading to genome instability should be carefully examined. Nuclease-based PE is often accompanied by the integration of the pegRNA scaffold and duplications of the HA sequence [221,277,278]. This is something rarely observed with a nickase-based PE approach, where a 1.7% average total insertion of any number of pegRNA scaffold nucleotides has been documented [187]. The difference is due to c-NHEJ precluding correct editing in the case of the nuclease-based approaches. While c-NHEJ inhibition with mutations or small molecules like DNA-PK inhibitors AZD7648, M3814 or NU7441 are effective in decreasing small c-NHEJ-mediated indels, they are also associated with an increased rate of genomic rearrangements [157,307,308].

Since a short-range PCR is commonly used to evaluate editing outcomes, such complex events are often overlooked, while the frequency of the detected alleles is overestimated. A recent study by Cullot et al. demonstrated that AZD7648 causes frequent kilobase-scale and megabase-scale deletions, chromosome arm loss and translocations when used for genome editing [309]. The kilobase-scale deletions can be partially prevented by simultaneous treatment with AZD7648 and a POL θ inhibitor, meaning that such deletions are caused by MMEJ [157,309]. However, MMEJ inhibition did not influence megabase-scale deletions [309], suggesting that another DSBR pathway may be responsible for their generation.

Another study suggests that SSA accounts for large deletions accumulating in cells with inhibited c-NHEJ [112]. Using a multi-pathway DNA repair reporter, which is able to discriminate between unmodified alleles, HR-repaired alleles, SSA-repaired alleles and small indels, van de Kooij et al. demonstrated that several tested DNA-PK inhibitors (NU7441, M3814 and AZD7648) led to a decrease in indels accompanied by a ~1.5–2.5-fold increase in both HR and SSA. The reporter system used in this study was, by design, prone to SSA due to the presence of long homologous sequences within 3 kb of the DSB. However, the data suggest that the same principle applies when long repeats are present at endogenous sites, such as at the *HBB* and *HBD* loci or abundant targets in the human genome flanked by *Alu* repeats [112].

Given that HR and SSA share the initial resection step it may be difficult to devise a strategy to selectively inhibit SSA without any effect on HR. Indeed, depletion of BRCA1 or short-range resection factors (CtIP, MRE11) led to a decrease in both HR and SSA, while depletion of BRCA2 inhibited HR but promoted SSA, consistent with previous findings on HR and SSA regulation [112]. Surprisingly, a knockdown of long-range resection nucleases selectively inhibited SSA. siRNA against *EXO1* demonstrated the most promising results, bringing SSA after NU7441 treatment to the level observed in cells that have not been treated with siRNAs and DNA-PK inhibitors. While SSA was decreased upon NU7441 + EXO1si treatment, HR was further promoted compared to cells treated with NU7441 + nontargeting siRNA [112].

Thus, while DNA-PK inhibition alone promotes large deletions and translocations, this effect may be partially prevented by using a combination of inhibitors targeting different DSBR pathways. These studies highlight the pressing need to investigate multiple potential editing outcomes using various techniques while continuing to explore DNA repair pathways and selective inhibitors. 

## Figures and Tables

**Table 1:** Summary of seven generations of prime editors. The characteristic feature of each editor differentiating it from previous generations is underlined.

Prime Editor	Cas9 Variant	Reverse Transcriptase	Guide RNA	Helper Protein
PE1 [187]	nSpCas9(H840A) nickase	wild-type M-MLV RT	pegRNA	-
PE2 [187]	nSpCas9(H840A) nickase as in PE1	M-MLV RT (D200N/L603W/T330P/T306K/W313F)	pegRNA	-
PE3 [187]	nSpCas9(H840A) nickase as in PE1	M-MLV RT (D200N/L603W/T330P/T306K/W313F) as in PE2	pegRNA + sgRNA complementary to the edited strand up- or downstream of the edit	-
PE3b [187]	nSpCas9(H840A) nickase as in PE1	M-MLV RT(D200N/L603W/T330P/T306K/W313F) as in PE2	pegRNA + sgRNA complementary to the edit established by the prime editor	-
PE4 [190]	nSpCas9(H840A) nickase as in PE1	M-MLV RT(D200N/L603W/T330P/T306K/W313F) as in PE2	pegRNA as in PE2	MLH1dn
PE5 [190]	nSpCas9(H840A) nickase as in PE1	M-MLV RT(D200N/L603W/T330P/T306K/W313F) as in PE2	pegRNA + nicking sgRNA as in PE3	MLH1dn
PE6a [192]	PEmax: nSpCas9(H840A) nickase with additional R221K and N394K substitutions	evo-Ec48: *Escherichia coli* Ec48 retron-derived RT with substitutions E60K/K87E/E165D/D243N/R267I/E279K/K318E/K343N evolved using PANCE	pegRNA as in PE2 or pegRNA + nicking sgRNA as in PE3	Optional: MLH1dn
PE6b [192]	nSpCas9(H840A) nickase with additional R221K and N394K substitutions as in PEmax	Evo-Tf1: *Schizosaccharomyces pombe* Tf1 retrotransposon-derived RT with substitutions P70T/G72V/S87G/M102I/K106R/K118R/I128V/L158Q/F269L/A363V/K413E/S492N evolved using PANCE.	pegRNA as in PE2 or pegRNA + nicking sgRNA as in PE3	Optional: MLH1dn
PE6c [192]	nSpCas9(H840A) nickase with additional R221K and N394K substitutions as in PEmax	PE6b with additional rationally designed substitutions (Tf1 RT P70T/G72V/S87G/M102I/K106R/K118R/I128V/L158Q/F269L/A363V/K413E/S492N/K118R/S188K/I260L/S297Q/R288Q)	pegRNA as in PE2 or pegRNA + nicking sgRNA as in PE3	Optional: MLH1dn
PE6d [192]	nSpCas9(H840A) nickase with additional R221K and N394K substitutions as in PEmax	M-MLV RT with the truncated RNaseH domain and additional mutations T128N/V223Y/D200C	pegRNA as in PE2 or pegRNA + nicking sgRNA as in PE3	Optional: MLH1dn
PE6e [192]	PEmax with additional substitutions K918A/K775R	M-MLV RT (D200N/L603W/T330P/T306K/W313F) as in PE2 but ΔRNaseH	pegRNA as in PE2 or pegRNA + nicking sgRNA as in PE3	Optional: MLH1dn
PE6f [192]	PEmax with additional substitutions E471K/H99R/I632V/H721Y/D645N/K918A	M-MLV RT (D200N/L603W/T330P/T306K/W313F) as in PE2 but ΔRNaseH	pegRNA as in PE2 or pegRNA + nicking sgRNA as in PE3	Optional: MLH1dn
PE6g [192]	PEmax with additional substitutions E471K/H99R/I632V/H721Y/R654C/D645N	M-MLV RT (D200N/L603W/T330P/T306K/W313F) as in PE2 but ΔRNaseH	pegRNA as in PE2 or pegRNA + nicking sgRNA as in PE3	Optional: MLH1dn
PE7 [191]	PEmax	M-MLV RT (D200N/L603W/T330P/T306K/W313F) as in PEmax	pegRNA as in PE2 or pegRNA + nicking sgRNA as in PE3	The N-terminal part of a small RNA-binding protein La(1–194) fused to the C terminus of PEmax. Optional: MLH1dn

## Data Availability

No new data were created.
